# Cardio-pulmonary exercise testing: An objective approach to pre-operative assessment to define level of perioperative care

**DOI:** 10.4103/0019-5049.68369

**Published:** 2010

**Authors:** Milind Bhagwat, Kaggere Paramesh

**Affiliations:** Department of Anaesthetics, Frimley Park Hospital, Frimley, Surrey GU16 7UJ, United Kingdom; 1 Kingston Hospital, Galsworthy Road, Kingston Upon Thames, Surrey KT2 7QB, United Kingdom

**Keywords:** Anaerobic threshold, cardiopulmonary exercise testing, congestive heart failure, cycle ergometer, exercise tolerance, functional capacity, postoperative mortality, preoperative assessment, VE/VCO_2_ slope, VO_2max_

## Abstract

Cardiopulmonary exercise testing is a non-invasive, objective method of assessing integrated response of heart, lungs and musculoskeletal system to incremental exercise. Though it has been in use for a few decades, the recent rise in its use as a preoperative test modality is reviewed. A brief account of cardiopulmonary exercise test, as it is carried out in practice and its applications, is given. The physiological basis is explained and relationship of pathophysiology of poor exercise capacity with various test variables is discussed. Its use for prediction of postoperative morbidity in various noncardiopulmonary surgical procedures is reviewed.

## INTRODUCTION

Cardiopulmonary exercise testing (CPET) is an objective method of determining an individual’s fitness for surgery. It provides an assessment of the integrative exercise responses involving the pulmonary, cardiovascular, haematopoietic, neuropsychological, and skeletal muscle systems, which are not adequately reflected through routine measurement of individual organ system functions.

This review article is aiming to describe the exercise physiology and principles behind the CPET, the practical way of conducting the test, explore the evidence and describe the interpretation of results relevant to anaesthetic practice. Although this test is also widely used by cardiologists, respiratory physicians and in sports medicine it is outside the scope of this article to cover those aspects.

## BACKGROUND

Currently, in majority of the hospitals in the United Kingdom, patients plan to have major operative procedures come for a nurse led pre-assessment clinic. They undergo various biochemistry, haematology and radiology investigations. Only a selected group of patients who satisfy the screening criteria are seen by an anaesthetist.

Majority organ function tests are carried out at rest and Treadmill exercise testing has low sensitivity for diagnosing coronary artery disease.[1] Currently grading patient risk of surgical morbidity is based on use of various scoring systems or classifications: ASA classification,[2] POSSUM score,[3] Lee’s Revised cardiac index,[4] etc. Integrating history of physical activity, signs and symptoms of individual organ function with above, gives stratified risk of perioperative morbidity. The process is subjective, variable with individual assessors and the tests performed do not look at whole body or its response to stress.

It is acknowledged that our current practice of preassessment is subjective. There is a possibility that some relatively fit patients may be wrongly allocated for postoperative care in the wards. This patient group may deteriorate or develop organ failure and eventually come to ICU. Similarly, some patients may be considered belonging to a high risk group and will be allocated to ICU postoperatively, using resources they may not need. If we rely on objective assessment, we not only use the resources appropriately but also reduce the morbidity and mortality by accurately predicting the required level of care in postoperative period.

Though fairly well-known in medicine, use of CPET as a tool in perioperative medicine was pioneered by older colleagues[5] in 1993. Since then, it is evolving as a preoperative assessment tool and importance of many variables measured during the test is revealing newer significance. CPET is gaining popularity in Europe for its objective nature and accurate assessment of postoperative cardiac morbidity. There is an established international society for exercise intolerance, research and education (www.iseire.org). It is involved in training and development of guidelines besides research in this field. A prospective trial of using CPET in predicting high risk surgical patient group who needs intensive care (ICU) or high dependency (HDU) care postoperatively has established the value of preoperative CPET.[6]

In the United Kingdom, improving surgical outcome group UK (ISOG UK) in its report for modernising care for patients undergoing major surgery, acknowledges the value of CPET and recommends use of CPET during pre assessment in hospitals doing major elective surgery.[7]

## PHYSIOLOGICAL BASIS

The energy for the exercising skeletal muscle comes from three sources. The immediate energy when muscle starts contracting comes from the stored energy within the muscle in the form of Creatine Phosphate. Before exhaustion of this energy source, the blood flow to the muscle increases due to increase in cardiac output. The next source of energy comes from aerobic metabolism of glucose, which incidentally is the efficient way of producing energy. Each molecule of glucose produces 37 ATP as energy during the aerobic phase. The increase in muscle workload is nicely matched by the stepped up function of cardio respiratory systems with rising delivery of oxygen and nutrients. With rising exercise load, this equilibrium of supply to demand increase only lasts for few minutes depending on efficiency of respiratory and cardiovascular systems in an individual. After few minutes of exercise the supply of oxygen to the muscle starts lagging behind the demand. This point also means the energy is not enough for the increasing exercise. The skeletal muscle cells, in addition to the aerobic metabolism for energy source are now resorting to the third source of energy the anaerobic metabolism. Anaerobic metabolism is very inefficient way of producing energy. Each molecule of glucose can only give 3 ATP as opposed to 37 in aerobic metabolism. The point at which anaerobic metabolism starts, is the point when cardio respiratory function lags behind to match oxygen demand. This point is called Anaerobic Threshold (AT) and expressed as oxygen consumption in ml /kg/min.

**Figure 1:**
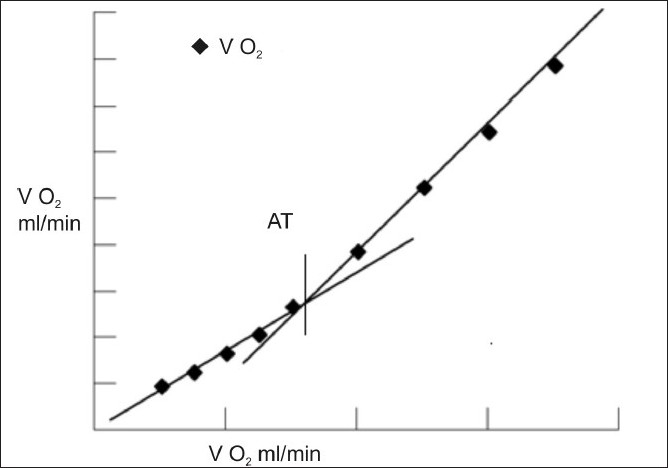
AT determination by Vslope

When the anaerobic metabolism starts providing energy, glucose is metabolised to lactic acid. The lactic acid being highly unstable non volatile acid with a pKa of 3.86, at body pH it dissociates to lactate^-^ and H^+^. The resulting acidosis helps oxygen extraction by tissues by favouring dissociation of oxygen from haemoglobin. The H^+^ is buffered by the cellular bicarbonate (HCO_3_^-^) to become carbon di-oxide (CO_2_) and water. Thus anaerobic metabolism creates an additional source of CO_2_. Thus, the amount of CO_2_ produced in the body is disproportionately higher than O_2_ consumed. By plotting the breath by breath analysis of oxygen consumption to carbon dioxide production, we can easily identify a patient’s anaerobic threshold [Figure 1].

The importance of AT in CPET is not just because it is the point of failure of cardio respiratory system to step up response, but also that this point is not detectable clinically as it is effort independent.

The patient can still go on to continue exercise, increase his/her aerobic and anaerobic metabolism and accumulates oxygen debt. Maximum oxygen uptake (VO_2max_) is the measure of a person’s ability to raise cardio respiratory capacity to the highest limit. In healthy individuals a plateau in VO_2_ may be seen near maximal exercise.

It is expressed as amount of oxygen used in ml /kg/min. This point may be correlated clinically with history of exercise tolerance and by expressing in metabolic equivalents (MET). One MET was estimated at 3.5 ml O_2_/min/kg. However, body composition may cause variation in this value. Byrne and colleagues[8] found the average resting metabolic rate (RMR or 1 MET) was 2.56 ± 0.40 ml O_2_/ min/kg. Duke activity index gives an estimate of oxygen consumption in METs associated with daily activities.

## CPET IN PRACTICE

In practice CPET requires:

- An exercise machine: cycle ergometer, treadmill, or an arm crank
- Computer programmed to increase workload as per ramp or step protocols
- Pneumotachographs to measure breath by breath gas volumes
- Gas analyser to accurately assess both qualitative and quantitative O_2_ and CO_2_ in each breath.
- Continuous 12 lead ECG, non-invasive BP and pulse oxymeter
- Appropriately trained personnel to conduct the test and to interpret the data
- Resuscitation facilities should there need arises

**Figure 2:**
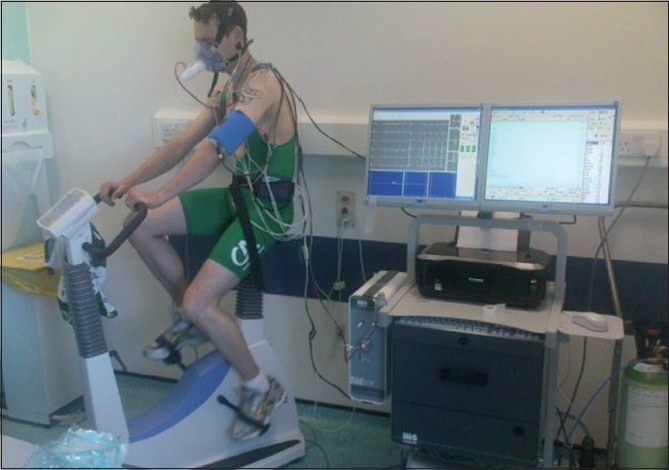
Bicycle ergometer

Most clinical systems rely on breath-by-breath analysis techniques because they provide the best measures of the metabolic response to exercise. Usually the exercise machine used in CPET is a cycle ergometer [Figure 2]. Patient sits on the ergometer and is connected to ECG, BP and SaO_2_. There will also be a tight fitting face mask which in turn is connected with in-line gas analyser. After 3 minutes of unloaded pedalling, the incremental ramp of load will start and is based on height, weight, sex and age of the patient.

**Figure 3:**
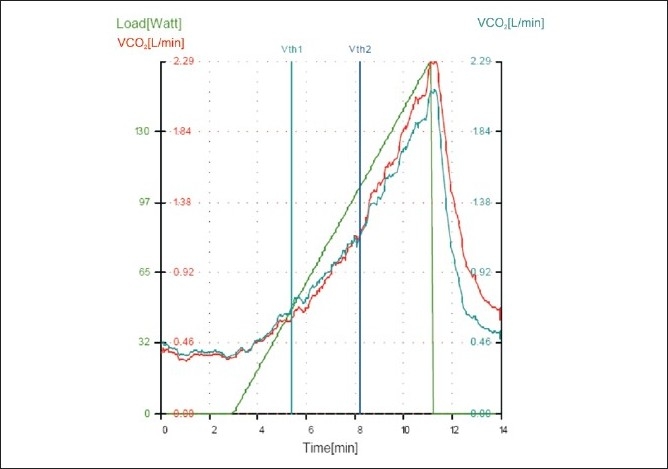
Breath by breath display of VCO_2_ and VO_2_

Graphs of breath by breath analysis of O_2_ and CO_2_ are continuously displayed and the point at which the CO_2_ starts increasing disproportionately to O_2_ is noted as the point of AT [Figure 3]. The patient will continue with exercise until getting exhausted or may reach a point of maximum oxygen consumption. The continuous ECG helps recognising any ischaemic changes and the test can be stopped.

### Evidence

Pioneering work by Mancini and colleagues[9] outlined use of VO_2peak_ as a survival marker in cardiac transplant patients. Beckles and colleagues[10] provided data to suggest VO_2max_ can be used to predict perioperative complication rates in tumour resection surgery.

However, the role of CPET in preoperative assessment of non-cardiothoracic surgical patients was established by Older and colleagues.

Older and colleagues,[5] published the CPET data on 187 elderly patients undergoing major operative procedures. They looked at the perioperative cardiovascular mortality and the relation to AT [Table 1]. They concluded that:

**Table 1:** Mortality data: AT above or below 11 ml/kg/min with preoperative ischaemia

AT ml/min/kg	No.	No. with ischaemia	CVS deaths	Mortality
<11	55	19	8	42
>11	132	25	1	4
Totals	187	44	9	(*P*<0.01)

- AT is unrelated to age of the patient.
- AT value of 11 ml/kg/min could be a discriminating point between relatively “fit” and “unfit” patients.

Combination of preoperative cardiac ischaemia and AT helps prediction of higher mortality

This was a pioneering work to assign patients objectively to high or low risk group.

**Figure 4:**
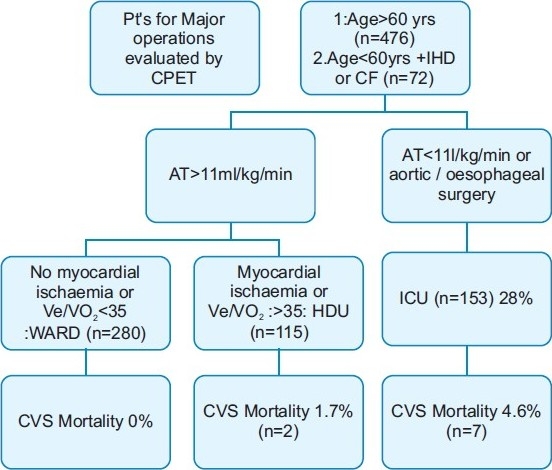
Use of AT and Ve/VO_2_ for postoperative management

Encouraged by this observation, Older and colleagues[11] used CPET on higher number of patients. They assigned the patients for postoperative management in ICU, HDU or wards according to the presence of ST changes in ECG, VE/VO_2_ ratio >35 and AT~11 ml/kg/min. As shown in Figure 4, correct utilisation of resources reduced cardiovascular mortality to 4.6% from 18%. Also, the patients allocated for postoperative care in the wards had 0% cardiovascular mortality.

There are more studies reported recently with similar prediction of mortality and the benefit of CPET in stratifying patients to postoperative care in ward, HDU or ICU.

Nugent and colleagues[12] followed abdominal aortic aneurysm (AAA) repair patients postoperatively up to 12 months. They did not find any correlation between preoperative VO_2peak_ and postoperative complications. This study had a small sample size of 30 patients and no information about power calculation is available. Carlisle and colleagues[13] measured AT, VO_2peak_, VE/VO_2_ and VE/VCO_2_ in 130 patients before elective repair of an AAA. In multivariate analysis VE/VCO_2_ correlated best with survival to both 30 days and the total observation period. AT was also a significant predictor in their study.

Foreshaw and colleagues[14] studied 78 patients undergoing oesophagectomy. They found low VO_2peak_ in patients who had postoperative cardiopulmonary complications.

McCullogh *et al*.,[15] studied the value of CPET in bariatric patients undergoing Roux-en-Y gastric bypass. They divided patients in tertiles depending on their VO_2peak_ values. Patients in the lower VO_2peak_ tertile had higher rate of complications and longer length of hospital stay. They didn’t investigate value of AT as an indicator of complications.

Nagamatsu and colleagues[16] observed patients undergoing oesophageal operative procedures for malignancy. They found that VO_2max_ was lower among patients having cardiopulmonary complications, though this was not seen for AT values.

## WHAT TO MEASURE

During CPET, in addition to continuous 12 lead ECG, SaO_2_ and BP, various measurements are obtained. In perioperative medicine following variables are most commonly used:

- Anaerobic threshold
- VO_2Max_ or VO_2Peak_ (maximum oxygen utilisation expressed as ml/kg/min)
- Ventilatory equivalent of O_2_ - V_eq_O_2_ (ventilation needed for taking up 1 litre O_2_)
- Ventilatory equivalent of CO - V_eq_CO_2_ (ventilation needed to remove 1 litre CO_2_)
- Oxygen pulse (Oxygen consumption/heart beat i.e. product of stroke volume and arterio-venous oxygen content difference)

The importance of additional variables is slowly emerging. Older used only the AT and Veq O_2_ as a guide to group the patients in different risk groups. They used V eq O_2_ (<35 L is considered normal) as a marker to identify patients with respiratory compromise.

AT and VO_2Max_ are used as markers of cardio-respiratory fitness of a patient, whereas value of Veq O_2_ and Veq CO_2_ as predictors of respiratory function is emerging. Many of the recent studies emphasize more on the predictive value of VO_2_ Max than the AT. A preoperative VO_2_ Max of >20ml/kg/min is considered reasonable for ward based care and >15 mls/kg/min may need vigilant postoperative care. VO_2Max_ <15ml/kg/min predicts poor prognosis.

Similarly values of <35L for Veq O_2_ and <42L for Veq CO_2_ are considered to be normal. A higher value suggests greater respiratory dysfunction. Recently there is emphasis on oxygen pulse which is VO_2_ /HR. It is important in prediction of probable cardiac dysfunction in patients with predicted poor AT. At rest usually oxygen pulse is 4-6 ml/heart beat. It indicates how well a patient’s cardiovascular system increases its capacity to provide for the increased demand. A good response is a continuous increase in oxygen pulse with work load initially. But this can reach plateau near maximum exercise as cardiac output in later stage of exercise depends only on heart rate but not on stroke volume.

## HOW TO USE THE TEST RESULTS?

In our hospital, all patients for elective major abdominal surgery over the age of 60 years attend pre assessment clinic to undergo CPET. Each patient will take 10-12 minutes for exercise protocol, however, the total test takes around 30-45 minutes including preparation. Those with unstable angina, fixed cardiac output states like severe aortic stenosis and those who cannot perform cycling are excluded. For postoperative care, patients are categorised in three groups: i) those considered fit for ward-based care postoperatively (AT >11, VO_2Max_ >15, Veq O_2_ <35, Veq CO_2_ <42, good increase in oxygen pulse from their base line); ii) those who may have an AT >11 but with cardiac ischaemia or abnormal ventilatory equivalents of either O_2_ or CO_2_ will go to HDU for postoperative care; iii) those who have AT <11, VO_2_ <15 or with other significant cardiac or pulmonary abnormality will go to ICU for postoperative care.

## CONCLUSION

In the presence of high demand for HDU and ICU facilities and the cost involved - appropriately using these resources is everyone’s responsibility. CPET will help us in better resource allocation by planning ICU/HDU management in perioperative period. Without CPET, pre-operative assessment tests were quite subjective in nature and were difficult to accurately identify patients who needed ICU after their operation. Though CPET is an old test, it is gaining its importance to bring accuracy to the assessment. As importance of variables other than AT and VO_2max_ is emerging, it will become a useful tool for all anaesthetists. We can also use CPET to provide precise information about risks involved and get information regarding consent of the patients.
